# Oroxin B Induces Apoptosis by Down-Regulating MicroRNA-221 Resulting in the Inactivation of the PTEN/PI3K/AKT Pathway in Liver Cancer

**DOI:** 10.3390/molecules24234384

**Published:** 2019-11-30

**Authors:** Nannan Li, Wenxiao Men, Yibo Zheng, Hechen Wang, Xiansheng Meng

**Affiliations:** 1 School of Pharmacy, Liaoning University of Traditional Chinese Medicine, Dalian 116000, China; linannan-999@163.com (N.L.); menwenxiao@hotmail.com (W.M.); g76543@sina.cn (Y.Z.); zakkwang@163.com (H.W.); 2 Component Medicine Engineering Research Center of Liaoning Province, Dalian 116000, China; 3 Liaoning Province Modern Chinese Medicine Research Engineering Laboratory, Dalian 116000, China; 4 Agilent Technologies Modern TCM and Multi-Omics Research Collaboration Lab, Liaoning University of Traditional Chinese Medicine, Dalian 116000, China

**Keywords:** Oroxin B, liver cancer, Microfluidic Chip, DEN-induced rats model, microRNA-221, PI3K/Akt/PTEN pathway

## Abstract

This study aims to investigate the anticancer effect of Oroxin B (OB) both in vitro and in vivo, and the molecular mechanism involved in microRNA-221 and the PI3K/Akt/PTEN pathway through modulation of apoptosis in Hepatocellular carcinoma (HCC). DEN-induced rats and HepG2 cells based on the microfluidic chip were employed, while the mRNA and protein expression of microRNA-221, PI3K, p-Akt and PTEN were evaluated by RT-PCR and Western blot analysis. Based on Microfluidic Chip and DEN-induced rat model, OB effectively exerts anti-liver cancer effect both in vitro and in vivo, and the expression of miR-221 in OB treated groups was significantly lower than that in the control group (** *p* < 0.01). The RT-PCR and Western blot results suggested the PI3K mRNA and protein in OB treated groups were both lower than those in control group and indicated the overexpression of PTEN. Therefore, OB effectively exerts anticancer effects by positively regulating the PTEN gene and then inactivating the PI3K/Akt signaling pathway through down-regulating the expression of the microRNA-221, thereby inducing apoptosis of liver cancer cells. This study offers a theoretical evidence for further development and clinical guidance of OB as an anti-tumor agent.

## 1. Introduction

Liver cancer, as a primary hepatic malignancy, is the second and sixth leading cause of cancer-related death to men and women, respectively. Meanwhile it is the fifth most common tumor worldwide [1]_._ The occurrence and development of liver cancer are related to many genes, including microRNAs. MicroRNA is a class of endogenous small RNA with a length of about 21–25 nucleotides [2]. It is reported that sustained down-regulation of microRNA-122 [3], microRNA-101 [4] and up-regulation of microRNA-221 (miR-221) [5] in the occurrence of liver cancer seemed to be particularly important. The oncogene microRNA, which is increasingly expressed in tumors, usually stimulate tumor development by negatively inhibiting tumor suppressor genes that control many biological processes, including cell proliferation, differentiation or apoptosis. As reviewed recently, miR-221, whose expression raises approximately 70%–80% in liver cancer [6], could affect several cancer related pathways by modulating multiple gene targets, including the pro-apoptotic protein B-cell lymphoma 2-modifying factor (BMF) [7], the inhibitor of the phosphoinositide 3-kinase pathway phosphatase and tensin homolog (PTEN) [8], ect. In addition to the PTEN gene, miR-221 can directly regulate it, thereby activating the PI3K/Akt signaling pathway to promote the invasion and metastasis of liver cancer [9]. Thses evidences on miR-221 strongly confirmed its critical role in liver cancer.

In recent years, traditional Chinese medicine (TCM) has achieved a good effect in improving the life quality of patients, and has drawn wide attention from the medical community. Oroxin B (OB, Baicalein7-*O-*β-gentiobioside, C_27_H_30_O_15_, MW 594.52 Da) is a flavonoid monomer compound of traditional Chinese medicine *Oroxylum indicum* (L.) Vent. Recent research shows OB have anti-lymphoma effect without obvious toxicity [10], and markedly inhibits the hemolytic activity of α-Hemolysin [11]. However, there are few reports on the anticancer effect and molecular mechanism of OB, and the previous studies in our laboratory showed that OB effectively exerts anticancer activity [12] and could down-regulated the expression of miR-221. Therefore, the purpose of this study was aimed to explore the anticancer effect of OB in vitro and in vivo and its possible molecular mechanism, in order to provide an experimental evidence for the development and application of OB as an anticancer agent.

## 2. Results

### 2.1. The Effect of Oroxin B on Proliferation of Human Hepatoma Cell Line HepG2

**Figure 1:**
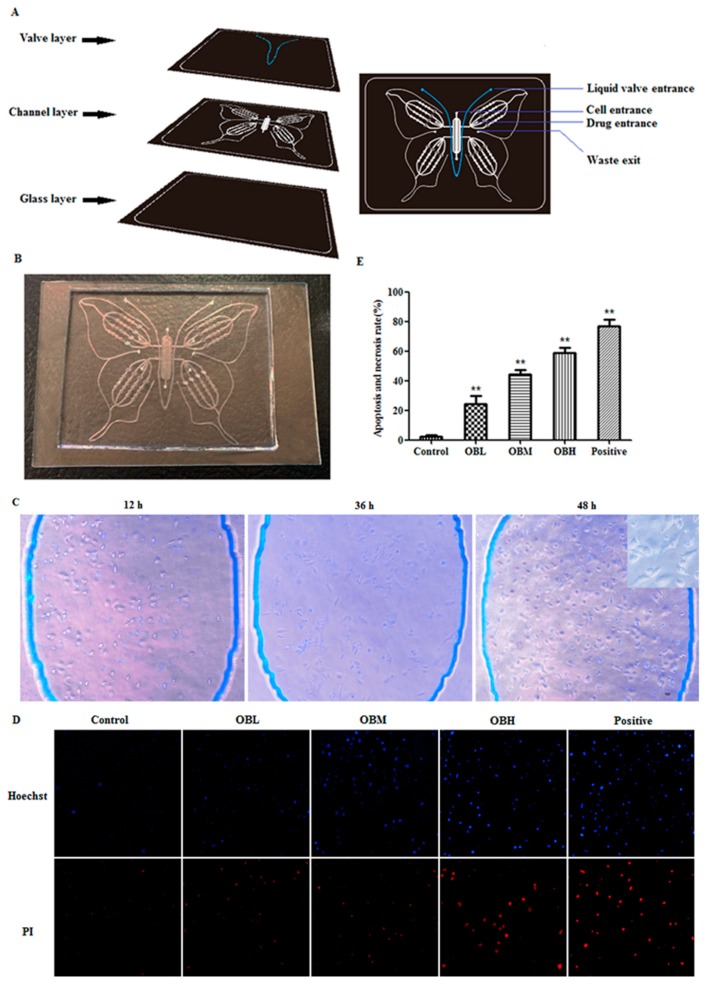
Schematic design of the Butterfly chip (**A**). The channel of blue was the valve layer, the white channels were PDMS fluid channel layer, the last black layer was glass for cell culture. The pictorial diagram of the Butterfly chip (**B**). Cell growth state in the Butterfly chip (**C**). The results of Hochest 33342/PI staining assay (**D**). Control group was untreated HepG2 cells; OBL represents OB low group (0.2 mg/mL); OBM represents OB middle group (0.4 mg/mL); OBH represents OB high group (0.6 mg/mL); Positive group was cyclophosphamide group; The histogram of apoptosis and necrosis rate (**E**). ** *p <* 0.01 vs. control group.

**Figure 2:**
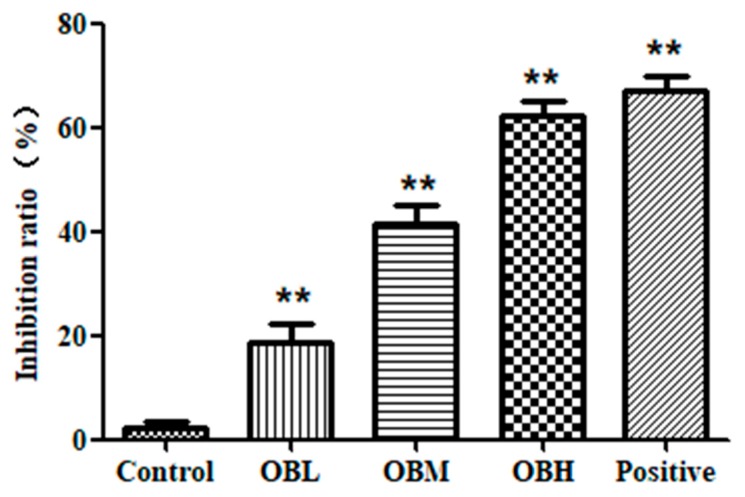
The inhibition ratio of HepG2 cells of MTT assay. ** *p <* 0.01 vs. control group.

HepG2 was cultured for 12 h, 24 h and 48 h in the Butterfly chip. As shown in Figure 1C, cells had a good survival rate in the chip. The result illustrated that cells were set up in a beneficial and stable system provided by the PDMS (polydimethylsilovane)-glass Butterfly chip, and could meet the experimental needs. After OB treatment, the Hochest33342/PI staining solution was utilized to detect the proliferation of HepG2 cells. As we all know, the apoptotic cells appear bright blue, and the necrotic cells appear bright red, as shown in Figure 1D–E, it is obvious that the necrosis and apoptosis rate of HepG2 cells in OB administration groups were higher vs. control group (*p* < 0.01), which illustrated the significant anticancer effect of OB in vitro. MTT assay was also used to verify the accuracy of the chip experiment results. From the results of the MTT assay (as shown in Figure 2), there was no significant difference between the results of MTT assay and the chip experiments. It was demonstrated that the chip experiment has high accuracy and feasibility.

### 2.2. General Appearance of Liver and Histopathological Evaluation

**Figure 3:**
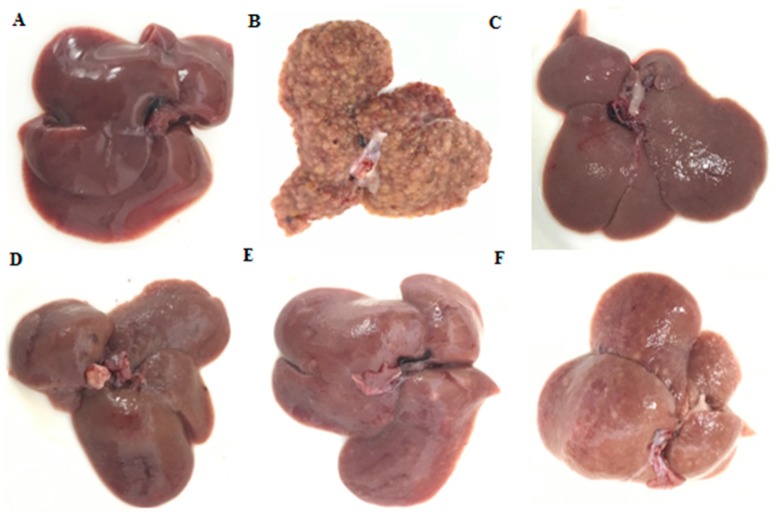
The general appearance of liver in each group. Blank group was not administered group (**A**), control group was DEN-induced group (**B**), positive group was cyclophosphamide group (**C**), OB high-dose group (**D**), OB medium-dose group (**E**), OB low-dose group (**F**).

**Figure 4:**
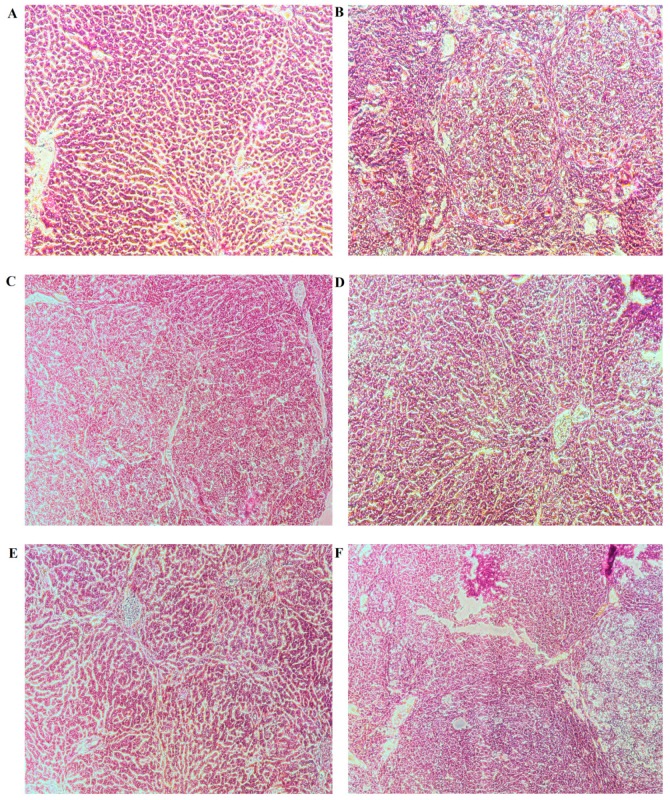
Histopathological examination of liver in each group. Blank group was not administered group (**A**), control group was DEN-induced group (**B**), positive group was cyclophosphamide group (**C**), OB high-dose group (**D**), OB medium-dose group (**E**), OB low-dose group (**F**). This tissues stained by H&E (100×).

From the general appearance of liver tissues in each group, at 16th week, nodules or tumors inducing by DEN (*N*-Nitrosodiethylamine) were visible to unaided or could be readily observed by unaided eyes (Figure 3B). All nodules with diameters above 1 mm were counted. Oral administration of OB before the occurrence of tumor could significantly inhibit its development. Compared with the control group (DEN-induced animals), OB treatment remarkably decreased the average number of nodules per liver to varying degrees in OB groups (Figure 3D–F). At week 16, H&E results showed the livers of the blank group animals remained normal tissue architecture. Meanwhile, architecture loss and neoplastic cells presented in the livers of control group. OB (24, 12 mg/kg) treated groups suggested significant improvement with near-normal architecture and fewer neoplastic cells (Figure 4).

### 2.3. The Effect of OB on the Levels of AFP and ALT in Serum of DEN-Induced Rats

**Figure 5:**
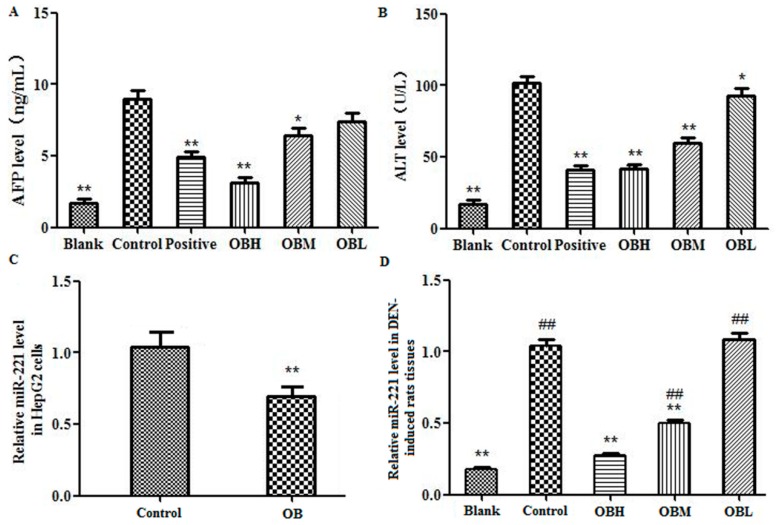
The levels of AFP and ALT in serum of DEN-induced rats. The concentration of AFP (**A**) and ALT (**B**) in the serum of DEN-induced rats. All data were expressed as mean ± SD, *n* = 6. * *p* < 0.05, ** *p* < 0.01. The miR-221 expression in hepatocellular carcinoma cells (**C**), control group was untreated HepG2 cells; OB was Oroxin B group; The micRNA-221 expression in DEN-induced rats liver tissues (**D**), blank group was not administered group, control group was DEN-induced group, OBH was OB high-dose group, OBM was OB medium-dose group, OBL was OB low-dose group. The levels of miR-221 was detected by RT-PCR and measured with U6 as an internal reference. All data were expressed as mean ± SD, *n* = 6. ** *p* < 0.01 vs. control group, ^##^ *p* < 0.01 vs. blank group.

ELISA assay was utilized to measure the levels of the tumor marker AFP (α-fetoprotein) and marker enzyme ALT (Alanine aminotransferase) which were markedly increased in the animal serum of control group (DEN-induced group) compared with that of the blank group (** *p* < 0.01). As shown in Figure 5A,B, compared with control group, the levels of AFP and ALT were significantly decreased (** *p* < 0.01 or * *p* < 0.05). Moreover, OBH group had no statistical significance in the comparison with the blank group *(p* > 0.05).

### 2.4. The Result of Microarrays

**Figure 6:**
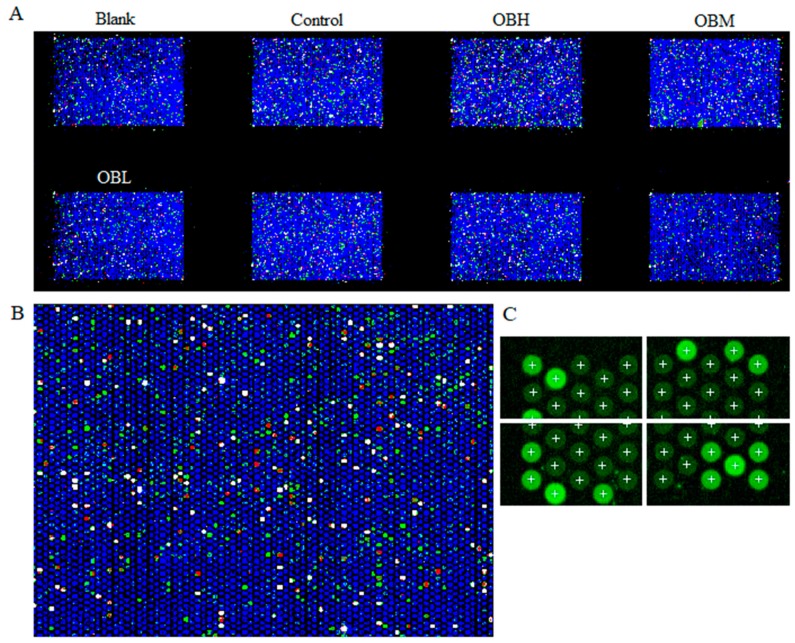
The result of images generated from scanned microarrays. Example of an image generated from a scanned eight-pack microarray (**A**). The result of partially magnifying the control group (**B**). Spot finding of the four corners of the array from control group (**C**). All data, repeated by three independent experiments.

An example of a scanned microarray is shown in Figure 6. As shown in Figure 6B, each circle represents a unique spotted probe. Red probes indicate that gene expression is higher than that in the control group after the OB treatment, while green probes indicate that gene expression is lower than in the control group. The data file was extracted with Agilent Feature Extraction 12.0.3.1 software and analyzed with Mass Profiler Professional software. *T*-test and log 2 Fold Change was performed to identify mircoRNAs whose expression was significantly changed after the OB treatment. The expression of 89 and 780 mircoRNAs was significantly altered by OB at the *p* < 0.05 and Fold change > 2 level, respectively. The partly significantly altered miRNA was shown in Table 1. Through references research, to find the genes which suggest greater correlation with liver cancer, finally miR-221 was determined for further study.

### 2.5. The Effect of OB on miR-221 Expression in HepG2 Cells and Liver Tissues

Real Time PCR was utilized to analyze the expression levels of miR-221 in cells and liver tissues. Results showed that the expression of miR-221 was significantly increased in both hepatocellular carcinoma cells HepG2 (control group) and DEN-induced rats (control group) liver tissues, indicating that miR-221 was involved in the progression of liver cancer. The expression of miR-221 in OB treated groups was significantly lower than that of the control group (** *p* < 0.01), as shown in Figure 5C,D.

### 2.6. The Effect of OB on PI3K, PTEN, Akt mRNA and Protein Expression in HepG2 Cells and Liver Tissues

**Figure 7:**
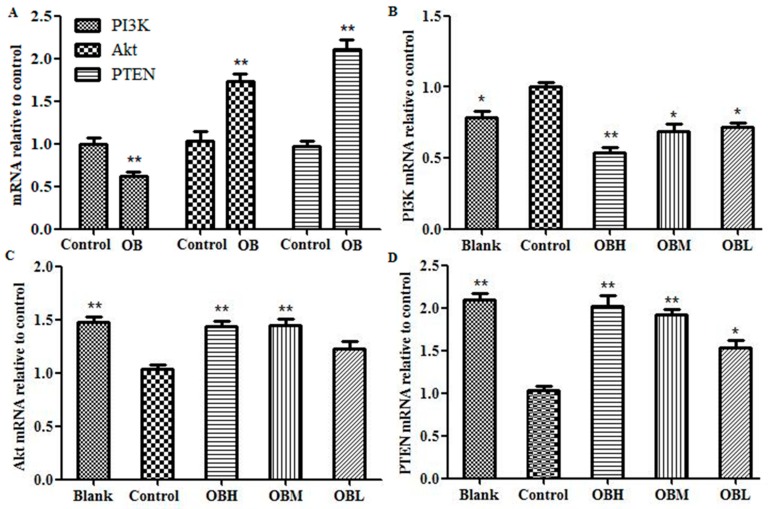
Results of PI3K, Akt and PTEN mRNA expression in HepG2 cells (**A**), control group was untreated HepG2 cells; OB was Oroxin B group. Statistical results of PI3K (**B**), Akt (**C**) and PTEN (**D**) mRNA expression in DEN-induced rats’ liver tissues, blank group was not administered group, control group was DEN-induced group, OBH was OB high-dose group, OBM was OB medium-dose group, OBL was OB low-dose group. The levels of mRNAs were detected by RT-PCR and measured with β-actin as an internal reference. All data, repeated by three independent experiments, are presented as mean ± SD. *p < 0.05 and ** *p* < 0.01 vs. control group.

**Figure 8:**
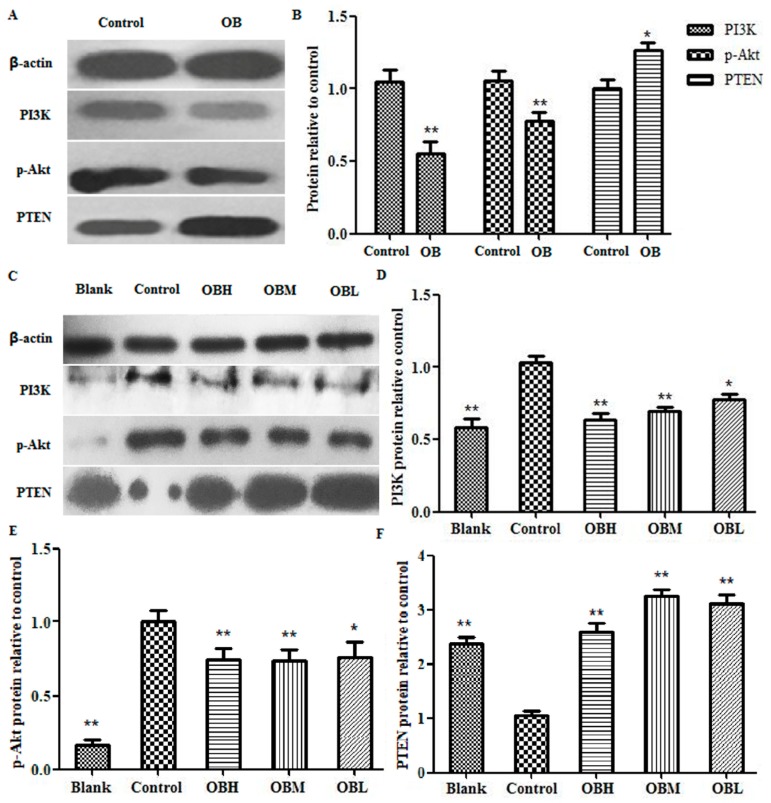
The results of PI3K, p-Akt and PTEN protein expression in HepG2 cells (**A**), Statistical results of PI3K, p-Akt and PTEN protein expression in HepG2 cells (**B**), control group was untreated HepG2 cells; OB was Oroxin B group. The results of PI3K, p-Akt and PTEN protein expression in DEN-induced rats’ liver tissues (**C**), Statistical results of PI3K (**D**), p-Akt (**E**) and PTEN (**F**) protein expression, blank group was not administered group, control group was DEN-induced group, OBH was OB high-dose group, OBM was OB medium-dose group, OBL was OB low-dose group. The levels of proteins were detected by Western blot method and measured with β-actin as an internal reference. All data, repeated by three independent experiments, are presented as mean ± SD. * *p* < 0.05 and ** *p* < 0.01 vs. control group.

The expression of PI3K, Akt, PTEN mRNA and protein both in HepG2 cells and DEN-induced rats’ liver tissues were detected by RT-PCR and Western blot assays. The RT-PCR results suggested the PI3K mRNA expression in OB administration groups were lower than that in control group both in vitro and in vivo, and the Akt and PTEN were overexpressed (Figure 7, * *p* < 0.05 or ** *p* < 0.01). In the results of Western blot experiment, when comparing the protein level with control group, PI3K and p-Akt were significantly down regulated, PTEN were up regulated in OB administration groups both in HepG2 cells and DEN-induced rats’ liver tissues (Figure 8, * *p* < 0.05 or ** *p* < 0.01).

## 3. Discussion

Microfluidic chip as one of the most popular cutting-edge technologies in the 21st century is widely used in the fields of life science, disease diagnosis [13] and treatment [14], drug screening [15], and so on. Research by Wang et al. [16] on human lung squamous carcinoma demonstrated that integrated microfluidic systems possessed many satisfactory advantages and could applied in cellular biological analysis. In the study of Xu et al. [17], for guide individualized treatment in lung cancer, a microfluidic chip was designed to test the chemotherapists’ drug sensitivity, and then was employed to screen the appropriate chemotherapy schemes. All evidence suggested the feasibility and advancement of microfluidic chip technology in the applications of cancer treatment and drug discovery. OB is one of the flavonoids compound in *Oroxylum indicum* [18]. Flavones have been approved to be effective constituents in *Oroxylum indicum*. Nagasaka et al. found that chrysin from *Oroxylum indicum* has anti-tumorigenic activity [19]. Wang et al. reported that Oroxylin A from *Oroxylum indicum* has anti-Allergic effect both in vivo and in vitro [20]. In our study, we found that the OB has excellent anticancer activity. In order to investigate the inhibitory effect of OB on hepatoma cells HepG2 in vitro, Butterfly microfluidic chip was used. There are 4 re-holes of each channel in the Butterfly chip for repeated test, therefore, the result comes out quickly and accurately by avoiding operating duplication and reducing operating errors. In addition, the continuous flow of culture and administration in vitro is analogous to the microenvironment of human body. The experimental results demonstrated that the microfluidic chip which we designed can be well applied to anti-cancer drug research.

It is crucial to create animal models in the research of liver cancer, chemically-induced models, genetically engineered transgenic models, genetically engineered knockout models, and diet-induced models are frequently used [21]. Among them, chemically-induced model is the most widely used. For investigating the ability to induce liver cancer, phytochemicals and antioxidants, such as dimethylnitrosamine (DMN), diethylnitrosamine (DEN), thioacetamide (TAA), aflatoxin and carbon tetrachloride (CCl4), were tested on rodent models [22], which was frequently employed, for the gene expression signature of DEN-induced rats was similar to that of human liver cancer [23]. Comprehensive considerate on multiple studies, we chose DEN-induced model to study the anti-liver cancer effect of OB in vivo. The results of liver general appearance, histopathological examination and the levels of AFP and ALT, indicated that OB had good anti-liver cancer activity in vivo, it is also shown that the study of OB as an anticancer drug is valuable and necessary.

**Figure 9:**
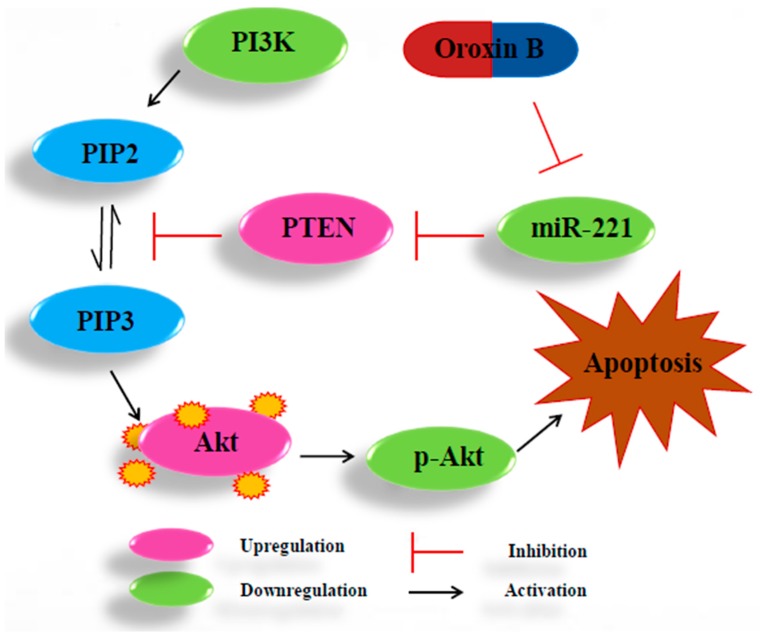
The relationship between miR-221 and PI3K, Akt, p-Akt, PTEN in HCC.

MicroRNAs (miRNAs) are a class of single stranded non-coding RNAs at the length of 22–25 nt [24]. miR-221 was reported to be overexpressed in several human malignant tumors, including liver cancer [25]. Rong et al. found that miR-221 overexpressed in hepatocellular carcinoma tissues, enhanced cell growth and inhibited apoptosis in vitro [26]. Wang et al. reported that overexpression of miR-221 could promote cell proliferation in human thyroid papillary carcinomas. The overexpression of miR-221 could participate in the processes of proliferation and apoptosis simultaneously, thus playing multiple roles in tumorigenesis [27]. A study by Zhao et al. suggested that miR-221 could bind to PTEN 3′-UTR to be a down regulator that inhibiting PTEN translation [28]. As one of the most commonly altered tumor suppressors, PTEN could convert phosphatidylinositol - 3, 4, 5-trisphosphate (PIP3) to phosphatidylinositol - 4, 5 - bisphosphate (PIP2), rise in protein synthesis, cell cycle progression, migration and survival, and thus inactivate PI3K/AKT pathway directly [29]. Zhang et al. reported that miRNA-221 could affect radiation sensitivity by regulating PTEN/AKT pathway in tumor cells [30]. Xie et al. found that miR-221 regulated cell proliferation and BCNU resistance in glioma cells by targeting the PI3K/PTEN/Akt signaling axis [31]. The relationship between miR-221 and PI3K, Akt, PTEN in liver cancer is shown in Figure 9. In present study, the results of qRT-PCR and Microarray scanner revealed that miR-221 expression markedly increased both in hepatoma HepG2 cells and DEN-induced rats liver tissues vs. control group, and OB administration could down-regulated the expression of miR-221 (as shown in Figure 5 C-D and Table 1). The results of RT-PCR and Western blot assays revealed that PI3K mRNA and protein expression were down-regulated and PTEN up-regulated after OB administration both in vitro and in vivo (as shown in Figure 7 and Figure 8). These findings demonstrated that OB could negatively regulate the PTEN gene and inactivate the PI3K/Akt signaling pathway through down-regulating the expression of miR-221, and thereby exerts the effect of anticancer. However, the fully mechanism of anticancer effect needs to be further studied.

## 4. Materials and Methods

### 4.1. Microfluidic Chip

The design and fabrication of the Polydimethylsiloxane-glass compound chip is completed independently by our laboratory. The Butterfly microfluidic chip consists of a valve layer and a channel layer of Polydimethylsiloxane (PDMS) (Midland, MI, USA), and a glass layer for cell culture. Oxygen plasma method was used for the irreversible chemical bonding of these three layers. The schematic diagram and physical map of the Butterfly chip were shown in Figure 1A,B.

### 4.2. Cell Culture

The hepatocellular carcinoma cell line HepG2 was obtained from Shanghai institute for biological sciences, Chinese academy of sciences institute of cell resource center (Shuanghai, China). HepG2 cells were cultured in DMEM medium (Gibco, MA, USA) with 10% FBS (Gibco, MA, USA), 100 U/mL penicillin-streptomycin solution in a 5% CO2, and incubator at 37 °C. When the cells were grown in logarithmic phase, 0.25% Trypsin-EDTA (Gibco, MA, USA) was used to digest it, the solution was prepared into single cell suspension using DMEM medium. Then, the single cell suspension was injected with precision syringe into the Butterfly chip for dynamic culture by peristaltic injection pump (Longer Pump, LSP04-1A, Baoding, China) at the flow rate of medium is 0.2 μL/min.

### 4.3. Proliferation Analysis

The proliferation analyses by Hoechst 33342/PI (Propidium Iodide) double staining kit (Solarbio, Beiing, China), the hepatoma cells were dynamic cultured in Butterfly chip and treated with OB (0.2, 0.4, 0.6 mg/mL) for 24 h, then Hoechst 33342 and PI were mixed at a volume ratio of 1: 1 in the dark, then injected into the Butterfly chip with precision syringe, staining 10 min in the dark at room temperature, and then, washed with 1×PBS 3 times. Finally, fluorescence was recorded on an inverted fluorescence microscope (Nikon, Japan) and take photos to calculate the cell apoptosis rate (%). Apoptosis and necrosis rate (%) = (Apoptotic cells + Necrotic cells)/Total cell × 100%.

### 4.4. MTT Assay

MTT assay was also used to determine cell viability in order to verify chip experiment results. Briefly, cells were plated in 96-well plates at a density of 1 × 10^4^ per well. After overnight culture, different concentrations of OB (0.2, 0.4, and 0.6 mg/mL) were added to the wells and cells were incubated for 24 h. Then add MTT (Sigma St. Louis, MO, USA) and continued to incubate for 4 h. Subsequently, added 150 μL DMSO (Sigma St. Louis, MO, USA) to each well. Absorbance was measured with a Spectra Max Plus microplate reader (Molecular Devices, CA, USA) at a wavelength of 492 nm.

### 4.5. Animals and Chemicals

Male SD rats weighing 180 to 220 g, were obtained from Liaoning Changsheng Biological Technology Co., Ltd. (Liaoning, China). All experiments were performed in accordance with the approved animal protocols and guidelines established by Medicine Ethics Review Committee for animal experiments of Liaoning University of Traditional Chinese Medicine, approval number: 2019YS(DW)-033-01. The animals were housed in polycarbonate cages and fed with commercial pellet diet and water *ad libitum*. The guidelines were established by the Liaoning University of Traditional Chinese Medicine and were approved by the university committee for animal experiments. Diethylnitrosamine (DEN) was purchased from Sigma Aldrich (St. Louis, MO, USA).

### 4.6. Animals Experimental

The animals were acclimatized to the laboratory conditions for approximately seven days before beginning the experiments. The rats were randomized into six groups and each group contained 30 animals, blank group (normal animals), control group (DEN induced group), OB high group (OBH, 24 mg/kg body weight/day), OB middle group (OBM, 12 mg/kg body weight/day), OB low group (OBL, 4 mg/kg body weight/day) and positive group (Positive, 10 mg/kg body weight/day cyclophosphamide). All five groups except the blank group were treated with DEN at 70 mg/kg body weight/week of intragastric for 16 weeks. Oroxin B and cyclophosphamide treatment were started at 6 weeks after the hepatocarcinogenesis induction and continued for 16 weeks. All rats were sacrificed after 16 weeks [32,33,34].

The 8–10 surviving rats in each group were sacrificed under anesthesia induced with 3% pentobarbital sodium. Serum was obtained from the blood by centrifugation at 3000 rpm for 15 min at 4 °C, and stored at −80 °C for ELISA analysis. The livers were resected after the rats were sacrificed, then the left lobe of each liver were fixed in 4% paraformaldehyde solution for histopathological examinations. The remaining livers were stored at −80 °C for RT-PCR and Western blot analysis.

### 4.7. Histopathology

The liver samples of each group were fixed in 10% formalin solution for over 24 h, followed by paraffin embedding, xylene dewaxing, alcohol dehydrating treatment, and sections cut at a thickness of 5 μm. Then, the sections were stained with hematoxylin and eosin (H&E). Liver pathological changes were examined using a microscope for histopathologial examination [35,36].

### 4.8. ELISA Assays of ALT, and AFP

The serum levels of ALT and AFP were measured using commercial kits (Lengton Bioscience Co., Ltd, Shanghai, China) according to the manufacturer’s instructions. The absorbance was measured with a Spectra Max Plus microplate reader (Molecular Devices, Sunnyvale, CA, USA) at a wavelength of 450 nm.

### 4.9. Microarray Analysis

Total RNA was extracted from liver tissue, using TRIzol (Ambion, Carlsbad, CA, USA) according to the manufacturer’s instructions. After phosphatase treatment, 100 ng total RNA for each sample was incubated for 30 min at 37 °C. The mixture with DMSO was incubated at 16 °C for 2 h for assemble labeling reaction. Samples were dried with vacuum concentrator, approximately 2 to 3 h at 55 °C, and then performed assemble hybridization. Arrays were hybridized at 55 °C for 20 h, 20 RPM. After hybridization, arrays were washed with Gene Expression Wash Buffer 1 and Gene Expression Wash Buffer 2 according to the manufacture’s protocols. Arrays were dried with airlaid paper and scanned with a Sure Microarray Scanner (Agilent Technologies, Santa clara, CA, USA). Data were extracted with Agilent Feature Extraction 12.0.3.1 software, and analyzed with Mass Profiler Professional software 12.6 (Agilent Technologies, Santa clara, CA, USA).

### 4.10. RNA Isolation and Quantitative RT-PCR

CDNA was synthesized from random hexamers and reverse-transcribed using TransScript One-Step gDNA Removal and cDNA Synthesis SuperMix (TransGen, Beiing, China). Gene expression levels were measured with RT-PCR using TransScript Top Greenq PCR SuperMix (TransGen, Beiing, China) in Piko Thermal Cycler 96-well system (Thermo, Waltham, MA, USA). Each sample was analyzed in triplicate. Relative levels of mRNA expression were normalized for β-actin mRNA expression and calculated according to the formula 2^−(ΔCt sample-ΔCt control)^. Primer sequences for the genes: PTEN F: 5′-CCCAGTCAGAGGCGCTATG-3′, R: 5′-GGCAGACCACAAACTGAGGATT-3′; Akt F: 5′-CTCATTCCAGACCCAGAC-3′, R: 5′-CAGCCCGAAGTCCGTTA-3′; PI3K F: 5′-AACGAGAACGTGTGCCATTTG-3′, R: 5′-AGAGATTGGCATGCTGTCGAA-3′; β-actin F: 5′-CACCCGCGAGTACAACCTTC-3′, R: 5′-CCCATACCCACCATCACACC-3′ (Invitrogen, Carlsbad, CA, USA). micoRNA quantification was determined by using Bulge-loop miRNA RT-PCR Primer Set (one RT primer and a pair of PCR primers for each set) specific for miR-221, designed by RiboBio (RiboBio Co. Ltd., Guangzhou, China). The U6 gene was used as an internal control.

### 4.11. Western Blotting Analysis

Total protein was extracted from liver tissue, using RIPA protein lysis buffer containing 1 mM PMSF. 300 ng total protein were separated by 10% SDS-PAGE gel at 80 V for 1 h, transfered with polyvinylidenedifluoride (PVDF) membrane at 100 V for 1.5 h, blocked in 5% BSA (Solarbio, Beijing, China) and probed with appropriate primary antibodies against the target proteins. PTEN (Cat:#9188S), PI3 Kinase p85 (Cat:#4292S) anti bodies were purchased from Cell Signaling (Beverly, MA, USA). p-Akt (Cat:66444-1-Ig) and β-actin (Cat:20536-1-AP) anti bodies were purchased from Proteintech (Protein tech, Chicago, IL, USA). These were followed by incubation with Goat Anti-Rabbit immunoglobulin (Ig) G (H+L) (Cat:SA00001-2) (Protein tech, Chicago, IL, USA), and antigen-antibody complexes were visualized using the chemilucent ECL (TransGen Biotech, Beijing, China) detection system. The densitometric analysis conducted by Image J software 1.6.0 (National Institutes of Health, Bethesda, MD, USA) [37,38,39]. 

### 4.12. Statistics Analysis

Statistical analysis was performed using *t*-test or one-way ANOVA with GraphPad Prism 5.01 (San Diego, CA, USA). The *p* values were considered statistically significant at *p* < 0.05, and that *p* < 0.01 for very significantly difference. All data are means ± standard deviation (SD) for at least three separated experiments.

## Figures and Tables

**Table 1:** The partly significantly altered miRNA.

Name	Log FC ([OB] vs. [Control])	Regulation	Active_Sequence
miR-128-3p	−2.63	Down	AAAGAGACCGGTTCACTGTG
miR-129-1-3p	−4.21	Down	CTTTTTGGGGTAAGGG
miR-221-3p	−2.93	Down	GAAACCCAGCAGACAATGTA
miR-300-3p	−2.98	Down	GAAGAGAGCTTGCCCTTG
miR-324-5p	−2.81	Down	ACACCAATGCCCTAGGG
miR-328a-3p	−2.93	Down	ACGGAAGGGCAGAGAGGGC
miR-335	−4.32	Down	ACATTTTTCGTTATTGCTCT
miR-127-3p	−3.57	Up	AGCCAAGCTCAGACGGAT
miR-212-3p	5.44	Up	TGGCCGTGACTGGA
miR-3099	2.69	Up	TCCCCAACCTCTTTCT
miR-30b-3p	3.47	Up	GACGTAAACATCCACATCCCA
